# Application and Comparative Evaluation of Fluorescent Antibody, Immunohistochemistry and Reverse Transcription Polymerase Chain Reaction Tests for the Detection of Rabies Virus Antigen or Nucleic Acid in Brain Samples of Animals Suspected of Rabies in India

**DOI:** 10.3390/vetsci5010024

**Published:** 2018-02-27

**Authors:** K. Nithin Prabhu, Shrikrishna Isloor, B. Hanchinal Veeresh, Doddamane Rathnamma, R. Sharada, Lekshmi J. Das, M.L. Satyanarayana, Nagendra R. Hegde, Sira Abdul Rahman

**Affiliations:** 1 Commonwealth Veterinary Association—Crucell Rabies Diagnostic Laboratory, Department of Microbiology, Veterinary College—Bengaluru, Karnataka Veterinary Animal and Fisheries Sciences University, Bengaluru 560024, India; nithinprabhuk@yahoo.com; 2 Department of Microbiology, Veterinary College—Bengaluru, Karnataka Veterinary Animal and Fisheries Sciences University, Bengaluru 560024, India; veereshhanchinal@gmail.com (B.H.V.); doddamanerathnamma@yahoo.co.in (D.R.); sharadadr@yahoo.co.in (R.S.); lekshmijdas10@gmail.com (L.J.D.); 3 Department of Pathology, Veterinary College—Bengaluru, Karnataka Veterinary Animal and Fisheries Sciences University, Bengaluru 560024, India; mlspathology@yahoo.com; 4 National Institute of Animal Biotechnology, Miyapur, Hyderabad 500049, India; hegde@niab.org.in; 5 Commonwealth Veterinary Association, Jayanagar, Bengaluru 560011, India; shireencva@gmail.com

**Keywords:** rabies, direct fluorescent antibody (DFA), direct rapid immunochemistry test (dRIT), reverse transcription polymerase chain reaction (RT-PCR), India

## Abstract

Accurate and early diagnosis of animal rabies is critical for undertaking public health measures. Whereas the direct fluorescent antibody (DFA) technique is the recommended test, the more convenient, direct rapid immunochemistry test (dRIT), as well as the more sensitive, reverse transcription polymerase chain reaction (RT-PCR), have recently been employed for the laboratory diagnosis of rabies. We compared the three methods on brain samples from domestic (dog, cat, cattle, buffalo, horse, pig and goat) and wild (leopard, wolf and jackal) animals from various parts of India. Of the 257 samples tested, 167 were positive by all the three tests; in addition, 35 of the 36 decomposed samples were positive by RT-PCR. This is the first study in which such large number of animal samples have been subjected to the three tests simultaneously. The results confirm 100% corroboration between DFA and dRIT, buttress the applicability of dRIT in the simple and rapid diagnosis of rabies in animals, and reaffirm the suitability of RT-PCR for samples unfit for testing either by DFA or dRIT.

## 1. Introduction

Rabies is an infectious viral disease that is almost always fatal following the onset of clinical symptoms. The disease causes about 59,000 human deaths annually worldwide, most of them being in Asia and Africa, particularly in resource-constrained countries [1]. Dog bites account for almost the entire incidence of human rabies, whereas rabies in animals could be attributed to a sylvatic cycle between wild as well as feral canines and other carnivores. Rabies is preventable, and domestic canine rabies has been eradicated or controlled in several countries by vaccination; however, sylvatic rabies still presents a danger in these countries [2,3,4]. Given the importance of the spread of rabies to humans by canids and other carnivores, the protracted incubation period, the often delayed appearance of typical symptoms, rapid and accurate diagnosis of rabies in animals is critical for prognostication, and for initiating and implementing post-exposure prophylaxis, infection-control strategies and public health measures. 

Since the recognition in the early 20th century of Negri bodies as being the pathognomonic histopathological lesion in brain or spinal-cord sections, an array of immunoassays and molecular techniques have been developed for the laboratory diagnosis of rabies [5,6,7,8]. Whereas Seller’s technique to visualize intracytoplasmic virus-inclusion body aggregates (Negri bodies) is simple and rapid, it is suitable only for fresh specimens. Paraffin-embedded tissue samples can be used for staining, but the method is time-consuming, and like Seller’s staining, lacks sensitivity. Comparatively, the detection of virus antigen or nucleic acid is both more sensitive and rapid. Antigen can be detected by various immunoassays such as the fluorescent antibody technique (FAT), enzyme-linked immunosorbent assay, immunochemistry (e.g., direct rapid immunochemical test or dRIT, indirect rapid immunochemistry test or IRIT), or immunoblot (immunochromatography, dot-blot). Among them, the direct fluorescent antibody (DFA) test is the gold standard [9,10]. This test detects the presence of rabies virus antigen in infected tissues, particularly in the brain. However, the higher cost involved in fluorescent microscopy, the requirement for specialized training, and its unsuitability for highly decomposed samples limit the wide usage of DFA in resource-limited countries. On the other hand, enzyme immunoassays, such as rapid rabies enzyme immunodiagnosis (RREID), are not only as sensitive and specific as FAT, but also can be applied to partially decomposed samples; however, brain tissues need to be homogenized, resulting in a potential exposure hazard to laboratory personnel. Of late, dRIT has been increasingly employed for the laboratory diagnosis of rabies, owing to its simplicity [11,12,13]. Here, tissue smears are fixed, reducing exposure hazard, and the result can be read by using a simple microscope within an hour. Although dRIT can perform as well as DFA, it cannot also be applied to samples in advanced stages of decomposition, a common occurrence in developing countries where maintaining the samples under a cold chain during transportation is a challenge. The latter obstacle can be overcome by the application of nucleic acid-detection techniques such as reverse transcription, followed by polymerase chain reaction (RT-PCR), nucleic acid sequence-based amplification (NASBA), or loop-mediated isothermal amplification (LAMP), which not only can be applied to decomposed samples, but are also more sensitive and specific than DFA and/or dRIT [14,15]; although the lack of standardized protocols and higher percentage of false positives are their disadvantages.

In this study, we compared the application of DFA, dRIT and RT-PCR for confirmatory diagnosis of rabies in suspected brain samples of animals resourced from different parts of India. Two hundred and fifty seven freshly collected and transported, or frozen (−80 °C) archived samples from cattle, buffalo, horse, goat, pig, dog, cat, jackal, leopard and wolf subjects were subjected to all the tests. We observed 100% concordance between DFA and dRIT, and also showed the utility of RT-PCR in detecting viral nucleic acid in a further 35 samples that were unsatisfactory for testing by both DFA and dRIT. The results support the utility of dRIT as a simple test that can be adopted to field conditions, and contribute to the epidemiology of rabies in India.

## 2. Materials and Methods

### 2.1. Samples

Samples (*n* = 257) were obtained during September 2012 to October 2014 from carcasses of animals presumptively diagnosed to have rabies based on symptoms. Some of the samples (*n* = 101; 51 from Kerala, 8 from Maharashtra, 19 from Punjab, 18 from Tamil Nadu, 5 from Uttar Pradesh) were sourced from other institutions, and were obtained for comparing the different diagnostic tests. These were archived samples that had been collected earlier based on presumptive diagnosis of rabies, and confirmed by DFA at laboratories located in the respective states. In case of samples (*n* = 156) sent to our laboratory for confirmation, whole intact brain or parts thereof had been submitted, based on the status of the animal at the time of post-mortem examination. For testing, either the cerebellum or the brain stem were used. The details of the samples are provided in Table 1.

### 2.2. Direct Fluorescent Antibody (DFA) Test

The DFA test was carried out as described previously [16]. As per the recommendations of the World Health Organization (WHO) and Office Internationale des Epizooties (OIE; World Organisation for Animal Health), all procedures with a potential risk of exposure of personnel to rabies virus were carried out in a laboratory with biosafety level 2 containment. Commercially available fluorescein isothiocyanate-conjugated anti-rabies monoclonal antibody (Merck Life Sciences Pvt Ltd., Mumbai, India) was used in the study. For titrating the conjugate, impressions of the brain stem were made from positive and negative archived samples confirmed previously by DFA, air dried for two minutes at room temperature (22–28 °C), fixed in chilled acetone at −20 °C for an hour, and air dried again at room temperature. Serial two-fold dilutions (1:10 to 1:320) of the conjugate stock (Light Diagnostics Rabies DFA III anti-nucleocapsid IgG-FITC conjugate, Merck Millipore, Temecula, CA, USA) in phosphate-buffered saline (PBS), pH 7.0, containing 0.0125% Evan’s blue (Sigma, Bengaluru, India), were applied to the slides and incubated for 30 min at 37 °C in a humidified chamber. After staining, the slides were wicked onto absorbent paper to remove excess conjugate, and rinsed by immersing in PBS for 3 to 5 min. The slides were blotted to remove excess liquid, then briefly air-dried, and visualized under a fluorescent microscope (Carl Zeiss AG, Göttingen, Germany). Each stained slide was read by three persons independently, and the consensus last dilution of the conjugate providing crisp and high fluorescent staining with minimal background was considered as the end-point dilution. The working stock was prepared as two dilutions lower than the end-point.

### 2.3. Direct Rapid Immunohistochemistry Test (dRIT)

The dRIT was performed at room temperature using the kit and the accompanying instructions provided by the Centers for Disease Control and Prevention, USA [17]. Slides containing the impressions were air-dried, fixed in 10% buffered formalin [(10 mL formalin (37–40% stock solution), 90 mL distilled water, 0.4 g NaH_2_PO_4_, 0.65 g Na_2_HPO_4_] for 10 min, dip-rinsed in PBS containing 1% Tween-80 (PBST), immersed in 3% hydrogen peroxide for 10 min, and dip-rinsed in fresh PBST. Excess liquid was removed after each rinse by blotting at the edges surrounding the impression. The slides were incubated in a humidified chamber with a cocktail of biotinylated mouse anti-rabies monoclonal antibodies (a kind gift from the Centers for Disease Control and Prevention (CDC), USA) for 10 min, dip-rinsed in PBST, incubated with streptavidin-peroxidase complex (Kirkegaard and Perry Laboratories Inc., Gaithersburg, MD, USA) for 10 min, and dip-rinsed in PBST. Chromogenic substrate was prepared by adding 1 mL of acetyl 3-amino-9-ethylcarbazole (AEC) (provided as part of the kit) to 14 mL of 0.1 mol/L sodium acetate, pH 5.5, and 0.075 mL of 3% hydrogen peroxide. The slides were incubated with the AEC peroxidase substrate for 10 min, and dip-rinsed in distilled water. They were then counterstained with 1:2 Gill’s hematoxylin (provided as part of the kit) for 2 min, and dip-rinsed in distilled water. Finally, the impressions were mounted with a water-soluble mounting medium (provided as part of the kit), and examined by light microscopy (Carl Zeiss AG, Göttingen, Germany).

### 2.4. Cells and Viruses

As negative controls for nucleic acid detection, classical swine fever virus (CSFV) was used. The virus was propagated in PK-15 cells (ATCC, Manassas, VA, USA). The cells were cultured in Dulbecco’s modified Eagle’s medium supplemented with 10% fetal bovine serum (Invitrogen, Bengaluru, India), 100 U/mL of penicillin and 100 μg/mL of streptomycin (Invitrogen). For virus titration, PK-15 cells were seeded into 96-well plates at 10^4^/well, incubated overnight at 37 °C, and then infected in five replicates with log-fold serial dilutions of infected cell-culture supernatants. Cytopathology was scored after 72 h, and the 50% tissue culture infective dose (TCID_50_) was calculated using the Reed and Muench method [18].

### 2.5. Isolation of RNA from Brain Samples

Total RNA was extracted from brain tissues with TRIzol^®^ reagent (Invitrogen, Bengaluru, India) following the manufacturer’s instructions, with slight modifications. Brain tissue (50–100 mg) was added to 1 mL of TRIzol^®^ reagent, homogenized by grinding between two sterile cotton swabs, and incubated for 5 min at room temperature. Then, 0.2 mL of chloroform (Sisco Research Laboratories Pvt Ltd., Mumbai, India) was added per each mL of TRIzol^®^, and vigorously mixed for 15 s, before incubating at room temperature for 2–3 min. The samples were centrifuged at 11,000× *g* for 15 min at 4 °C. The aqueous phase was transferred to a fresh tube, and RNA was precipitated by mixing with isopropyl alcohol at 0.5 mL per mL of TRIzol^®^ used. The sample was incubated at room temperature for 10 min, centrifuged at 11,000× *g* for 10 min at 4 °C, and the RNA pellet was washed once at 4 °C with 1 mL of chilled 75% ethanol per mL of TRIzol^®^ used. The sample was mixed by vortexing and centrifuged at 6000× *g* for 6 min at 2–8 °C. The RNA pellet was resuspended in 80 μL of RNase-free water (Bangalore Genei Pvt Ltd., Bengaluru, India), and heated to 56 °C for 6 min, and then stored at −80 °C.

### 2.6. Reverse Transcription-Polymerase Chain Reaction (RT-PCR)

For the RT-PCR studies, a confirmed RABV isolate (VMC-KAR-05), obtained as a part of an earlier study [19], was used as the positive control. For negative control, a known healthy brain sample, and culture supernatants of cells infected with CSFV, were used. For the latter, PK-15 cells were infected at 0.1 TCID_50_/cell, and harvested when 80–90% cytopathology was observed. The culture supernatant was directly used in RT-PCR without titration to confirm the presence of CSFV nucleic acid (data not shown).

The cDNA synthesis was done using a High Capacity cDNA Reverse Transcription kit (Invitrogen), as per the manufacturer’s protocol, with slight modifications. The RT master mix was prepared by adding 2.0 μL of 10× RT buffer, 0.8 μL of 25× dNTP Mix (100 mM), 1.0 μL of MultiScribe™ (Thermo Fisher Scientific, Waltham, MA, USA) reverse transcriptase, 1.0 μL of RNase inhibitor, and 3.2 μL of nuclease-free water. This was added to 10 μL of RNA template and 2 μL (20 pmols) of JW12 primer [20], mixed and preheated at 94 °C for 1 min, and snap-cooled on ice for 5 min. Reverse transcription was carried out at 37 °C for 120 min, and a fragment of the N gene was amplified by PCR, as described previously [20], using the primers JW12 (5′-ATGTAACACCTCTACAATG 3′) and JW6(DPL) (5′CAATTCGCACACATTTTGTG3′) [20], which were obtained commercially (Eurofins Genomics Pvt. Ltd., Bengaluru, India). The PCR mixture comprised of 200 ng (3 µL) of cDNA, 2.0 µL (20 pmol) of JW12 forward, and 2.0 µL (20 pmol) of JW6 (DPL) reverse primers and 1 µL (100 µM) of each dNTP, 2.5 µL of 10X reaction buffer, 0.5 µL (1.5 U) of *Taq* DNA polymerase, and water to make up the volume to 25 µL. The DNA was denatured initially at 94 °C for 5 min, followed by 40 cycles of denaturation at 94 °C for 30 s, annealing at 50 °C for 30 s and an extension at 72 °C for 60 s, and a final extension of 10 min. The PCR products were analysed by 2% agarose gel electrophoresis in comparison with a 100 bp DNA ladder, and visualized using a gel documentation system (Bio-Rad Laboratories, Hercules, CA, USA).

## 3. Results and Discussion

Despite an estimated 35% of all the human rabies deaths worldwide occurring in the country [21,22], the disease is not notifiable in India. The lack of reporting is compounded by fear of touching cadavers, constraints in transporting the samples, and the availability of a limited number of laboratories capable of carrying out definitive diagnostic tests. As elsewhere, the major route of transmission of rabies virus to humans are dog bites, which constitute 91.5% of all animal bites in India [21]. More than 96% of rabies cases in India are the result of contact with infected dogs [23]. It has been estimated that India has one dog for every 36 persons, and the majority of these dogs are free=ranging or feral [23]. In addition, rabies has also been reported to have been contracted through contact with infected jackals, cats, monkeys, mongooses and foxes [24]. Thus, rabies in animals is not only a major concern for India, but also presents an opportunity for rapid action on post-exposure prophylaxis for humans, livestock and pets if it can be diagnosed quickly and easily. However, capacity-building in implementing validated or well established diagnostic tests and in instituting referral laboratories has been slow. In this context, an OIE twinning programme has recently been initiated at our rabies laboratory in Bengulugu. The work described here is part of a rabies diagnosis programme supported by Crucell and led to the development of the twinning programme [25].

**Figure 1:**
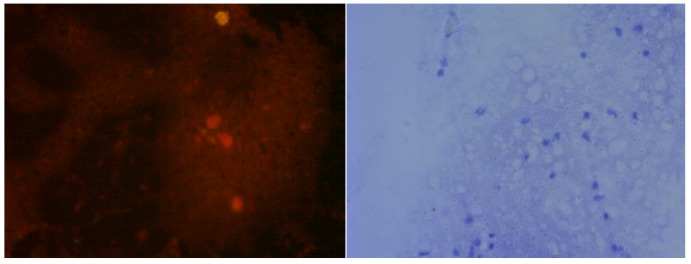

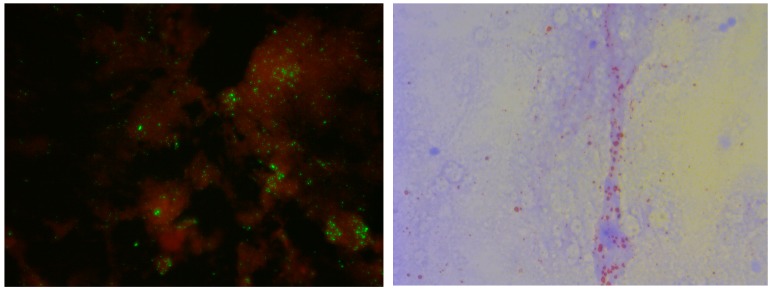
Brain impression from a non-rabid dog (**top two panels**) or a dog suspected of rabies (**bottom two panels**), subjected to direct fluorescent antibody (DFA) test using anti-rabies virus nucleocapsid protein IgG-FITC conjugate (**left two panels**) or to direct rapid immunochemistry test with (dRIT) using biotinylated mouse anti-rabies monoclonal antibodies and streptavidin-peroxidase, with hematoxylin couterstain (**right two panels**). Scale: 200×.

DFA is the test of choice for the laboratory confirmation of rabies [26]. In the current study, positive samples showed bright green fluorescent foci of varying size scattered within the smear, sometimes being clearly visible within neurons (Figure 1, left panels), mirroring similar descriptions by others [11]. However, DFA has several drawbacks such as the need for an expensive fluorescent microscope, well-trained personnel, and quality controlled reagents (antibodies, conjugates), and varied parameters used during microscopy, and incubation times and temperatures, not to mention the subjectivity in interpretation of the test results [27,28,29,30]. In addition, acetone used as fixative in DFA does not completely inactivate the virus, as demonstrated by the infectivity of acetone-fixed tissue for neuroblastoma cells [31], posing a potential biohazard to laboratory personnel. Indeed, complete inactivation of cell culture-derived rabies virus appears to require >30% acetone [32].

Some of the limitations of DFA can be overcome by dRIT. Whilst excellent concordance between dRIT and DFA has been observed with freshly prepared samples, dRIT could perform better than DFA with frozen or fixed samples [11,12,33,34]. Positive results with dRIT can be declared by the presence of dark red- to brown-colored deposits scattered throughout the impression (Figure 1, right panels), as has been shown earlier [34,35]. Immunohistochemistry tests have been found to be as reliable as DFA for confirming rabies using tissues obtained from various animal species as well as those inoculated experimentally, even when the tissues had been stored frozen for various lengths of time and/or fixed [36,37]. Another advantage of dRIT is the use of formalin for fixing the tissue smears. Titres of cell culture-derived virus have been shown to be reduced by three orders of magnitude with 3–4% formaldehyde treated for 30 min [32], and complete inactivation can be achieved with 10% formaldehyde treatment for as little as 11 min [38], although it is arguable that cell culture-derived and tissue-embedded virus could be differentially affected by the same treatment.

The dRIT typically uses monoclonal antibodies (MAbs) to detect rabies virus antigen. However, it is possible that there could be slight variation in the amino acid sequence of the N protein targeted by these MAbs, resulting in varied sensitivity and specificity of the assay. In addition, variability in the quality of conjugates could also influence the assay sensitivity, potentially leading to inconclusive results [29]. Polyclonal antibodies have been recently proposed as an alternative, and shown to have slightly higher sensitivity and specificity in detecting the antigen [12,39]. It might, therefore, be necessary for OIE reference laboratories to produce and distribute standard reagents for use by any laboratory worldwide. Alternatively, an assay using conjugated secondary antibodies [40] may be explored.

**Figure 2:**
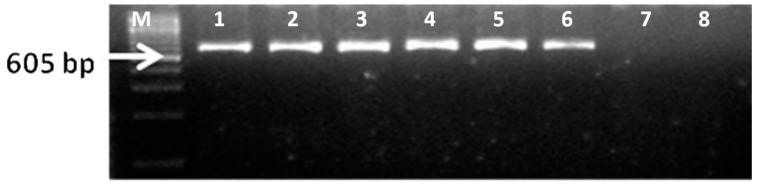
Polymerase chain reaction (PCR) based confirmation of RABV from suspected brain samples with JW12 and JW6deg primers (605 bp) (from different states). Lane M: 100 bp DNA ladder; Lane 1: VMC-147-Wolf-Karnataka; Lane 2: VMC-166-Dog-Kerala; Lane 3: VMCG-18-Dog-Tamil Nadu; Lane 4: VMC-86-Cattle-Andhra Pradesh; Lane 5: VMC-256-Cattle-Gujarat; Lane 6: Positive control; VMC-KAR-05; Lane 7: No template control; Lane 8: Negative control (CSFV cDNA).

A simple conventional RT-PCR has been found to be highly congruent to DFA in declaring positivity for rabies [35,41,42]. An example of the RT-PCR profile is depicted in Figure 2. The positive control yielded the expected amplicon of 605 bp, whereas no amplification was seen in negative or no template controls (NTC).

Analysis of data (see Table 2) from our studies (see Table 2) revealed that 1/1, 3/5, 92/144, 18/51, 8/9, 1/1, 1/1, 17/19, 3/3, 18/18 and 5/5 samples from Andhra Pradesh, Gujarat, Karnataka, Kerala, Maharashtra, Manipur, Pondicherry, Punjab, Rajasthan, Tamil Nadu and Uttar Pradesh, respectively, were positive by both DFA and dRIT, amounting to 167/257 (64.98%) of all the samples tested being positive by both the tests. It is to be noted that there was 100% agreement between the two tests in interpreting the results as positive, negative or inconclusive. Thirty six of the 257 samples (Table 2) had decomposed to various levels, and consequently produced considerable levels of non-specific auto-fluorescence in DFA (data not shown), making the interpretation ambiguous. This is in line with the earlier findings that there is a considerable loss of sensitivity of DFA when decomposed tissues are subjected to testing [14,42].

All of the 167 brain samples positive by DFA and dRIT were positive by RT-PCR (Table 2). In addition, 35 of the 36 samples, which were deemed to be unfit for either DFA or dRIT due to decomposition of the tissue, also yielded the expected amplicons by RT-PCR. Indeed, RT-PCR can detect the presence of nucleic acid in samples in decomposing conditions and collected several days earlier, transported at ambient temperatures, archived in frozen or fixed condition for several years, exhumed bodies, or in some cases, as an intravitam diagnostic assay [42,43,44,45,46,47,48,49,50,51,52]. This is not surprising since nucleic acids, especially fragments, are likely to be more resistant to tissue decomposition than proteins. This is of practical significance as delayed or improper transportation is often the cause of deterioration of clinical samples, leading to the inability to interpret the results or provide an indeterminate result. It should be noted, however, that a product of 605 bp, as amplified in our study, could miss some samples as the target region is more susceptible to fragmentation and/or degradation as compared to a smaller amplicon.

## 4. Conclusions

The ASSURED (affordable, sensitive, specific, user-friendly, rapid, equipment-free, door-step) criterion is especially applicable for the diagnosis of rabies since the disease is invariably fatal and occurs in resource-limited countries. The dRIT is a simple test for the diagnosis of rabies, and could be considered in place of the current standard test, the DFA. However, requirement for a microscope and trained personnel, as well as refrigeration of reagents is still an impediment to the widespread use of dRIT. Various laboratories have, therefore, developed immuno-chromatographic dip-stick tests using lateral flow assays for the diagnosis of rabies as well as other related lyssaviruses [53,54,55,56,57,58,59,60,61]. However, these assays need a lot of standardization since a wide range of sensitivities, specificities and batch-to-batch variation have been observed when compared side by side [57,62], and incomplete inactivation of the virus could be a biohazard [61]. In addition to lateral flow tests, nucleic acid-based assays which are suitable for field use, such as the loop-mediated or other isothermal amplification techniques [63,64,65], hold promise for the diagnosis of rabies, despite challenges in the standardization and validation of these molecular tests [66]. Table 3 presents a comparison of the various tests developed or employed for the diagnosis of rabies.

## Figures and Tables

**Table 1:** Details of samples collected/resourced.

		State or Union Territory										
	Andhra Pradesh *	Gujarat	Karnataka	Kerala	Maharashtra	Manipur	Pondicherry	Punjab	Rajasthan	Tamil Nadu	Uttar Pradesh	Total
Dog	1	-	126	45	9	1	1	6	3	17	2	211
Cattle	-	3	9	4	-	-	-	4	-	-	1	21
Buffalo	-	1	1	-	-	-	-	6	-	-	2	10
Cat	-	1	3	1	-	-	-	-	-	-	-	5
Horse	-	-	3	-	-	-	-	1	-	1	-	5
Pig	-	-	-	-	-	-	-	1	-	-	-	1
Goat	-	-	-	1	-	-	-	-	-	-	-	1
Jackal	-	-	-	-	-	-	-	1	-	-	-	1
Leopard	-	-	1	-	-	-	-	-	-	-	-	1
Wolf	-	-	1	-	-	-	-	-	-	-	-	1
TOTAL	1	5	144	51	9	1	1	19	3	18	5	257

* includes samples from both the newly carved out states of Telangana and Andhra Pradesh.

**Table 2:** Details of the results of DFA, dRIT and reverse transcription polymerase chain reaction (RT-PCR).

Species	Samples Collected	Positive by DFA	Positive by dRIT	Positive by RT-PCR	Unfit for DFA/dRIT	Negative
Dogs	211	136	136	168	32	42
Cattle	20	16	16	16	1	6
Buffalo	10	10	10	10	0	0
Cats	5	0	0	2	2	3
Horses	5	2	2	2	0	3
Pig	1	1	1	1	0	0
Goat	1	0	0	1	1	0
Jackal	1	1	1	1	0	0
Leopard	1	0	0	0	0	1
Wolf	1	1	1	1	0	0
**TOTAL**	**257**	**167**	**167**	**202**	**36**	**55**

**Table 3:** Comparison of DFA, dRIT, RT-PCR and the immunochromatrographic test (ICT) for the laboratory diagnosis of rabies.

	DFA	dRIT	RT-PCR	ICT
Equipment required	Fluorescent microscope	Light microscope	Thermal cycler	None
Fixative used	Acetone	Formalin	Various	None
Inactivation of the virus	Incomplete	Yes	Dependent on method	Incomplete
Applicability to decomposed tissue	No	No	Yes	Not always
Requirement for further standardization and validation	No	No	Yes	Yes
